# The Investigation on Ultrafast Pulse Formation in a Tm–Ho-Codoped Mode-Locking Fiber Oscillator

**DOI:** 10.3390/molecules26113460

**Published:** 2021-06-07

**Authors:** Jingcheng Shang, Yizhou Liu, Shengzhi Zhao, Yuefeng Zhao, Yuzhi Song, Tao Li, Tianli Feng

**Affiliations:** 1 China Key Laboratory of Laser & Infrared System (Ministry of Education), Shandong Provincial Key Laboratory of Laser Technology and Application, School of Information Science and Engineering, Shandong University, Qingdao 266237, China; 201920403@mail.sdu.edu.cn (J.S.); yizhou.liu@sdu.edu.cn (Y.L.); shengzhi_zhao@sdu.edu.cn (S.Z.); litao@sdu.edu.cn (T.L.); 2 Collaborative Innovation Center of Light Manipulations and Applications, School of Physics and Electronics, Shandong Normal University, Jinan 250358, China; yuefengzhao@sdnu.edu.cn (Y.Z.); yzsong@sdnu.edu.cn (Y.S.)

**Keywords:** nonlinear polarization rotation, mode-locking, ultrafast fiber oscillator, soliton pulse, noise-like pulse, Tm–Ho-codoped fiber

## Abstract

We experimentally investigate the formation of various pulses from a thulium–holmium (Tm–Ho)-codoped nonlinear polarization rotation (NPR) mode-locking fiber oscillator. The ultrafast fiber oscillator can simultaneously operate in the noise-like and soliton mode-locking regimes with two different emission wavelengths located around 1947 and 2010 nm, which are believed to be induced from the laser transition of Tm^3+^ and Ho^3+^ ions respectively. When the noise-like pulse (NLP) and soliton pulse (SP) co-exist inside the laser oscillator, a maximum output power of 295 mW is achieved with a pulse repetition rate of 19.85-MHz, corresponding to a total single pulse energy of 14.86 nJ. By adjusting the wave plates, the fiber oscillator could also deliver the dual-NLPs or dual-SPs at dual wavelengths, or single NLP and single SP at one wavelength. The highest 61-order harmonic soliton pulse and 33.4-nJ-NLP are also realized respectively with proper design of the fiber cavity.

## 1. Introduction

**Figure 1:**
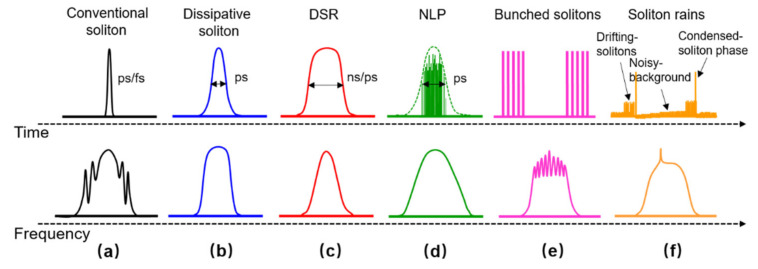
The typical pulse shapes and spectral profiles for conventional soliton (**a**), dissipative soliton (**b**), dissipative soliton resonance (DSR) (**c**), noise-like pulse (NLP) (**d**), bunched solitons (**e**), and soliton rains (**f**).

With the rapid development of ultrafast fiber lasers, increasingly complex ultrafast dynamics are discovered. The investigation of different ultrafast dynamics not only helps toward better understanding of the pulse evolution in an optical fiber, but also is useful for designing a mode-locking oscillator. Various ultrafast pulse evolution dynamics can be investigated theoretically based on the nonlinear Schrödinger equation [1,2], the coupled/complex Ginzburg-Landau equations [3,4], the Hirota bilinear formula [5], the Bogoyavlenskii–Schiff equation [6], and the Hirota–Satsuma-Ito equation [7]. The formation of soliton pulse is a well-known ultrafast dynamics which arises from the balance between the optical nonlinearity and anomalous chromatic dispersion [8,9,10,11]. The soliton pulse (SP) maintains its shape in both temporal (ps or fs) and spectral domain when propagating inside the fiber oscillator. The symmetrical Kelly sidebands distributed on the emission spectrum is a typical characteristic of the soliton pulse (see Figure 1a). In comparison, dissipative soliton is usually realized in the normal dispersion regime, always featured with a ps-long Gauss or sech shape in time domain and a square spectrum shape in frequency domain (see Figure 1b) [12,13,14]. Both of these two basic soliton pulses, SP and dissipative soliton, can turn into the dissipative soliton resonance (DSR) if large identical dispersion and high gain are simultaneously introduced into the mode-locking oscillator [15,16,17,18]. The DSR is characterized with a square pulse shape with an ns- or ps-pulse duration, smooth spectrum profile and large pulse energy (see Figure 1c). Furthermore, the basic soliton pulse will break into multiple pulses when the intracavity nonlinearity is overdriven by a large energy pulse. According to the pulse profiles in time domain, the pulse can be divided into different types: noise-like pulse (NLP) [19,20,21,22,23], bunched soliton pulses/optical soliton molecules [24,25,26,27] and soliton rain [28], et.al. NLP consists of a large number of small pulses randomly underlying in the same pulse envelope. Its spectrum is smooth without any spikes or modulations (see Figure 1d). Bunched soliton pulses also referred as optical soliton molecules are formed by multiple pulses gathered equal temporal distance. The typical characteristic of bunched soliton pulse is the interference fringes on the top of the spectrum (see Figure 1e). Soliton rain comprises three parts: a high peak pulse called condensed soliton phase similar to NLP with a group of multiple pulses under the envelope, the drifting pulses named drifting solitons emerging from the noise background and vanishing until reaching the condensed soliton phase, a wide noise background which manifests its existence as a small peak on the top of the spectrum in the frequency domain (see Figure 1f). These different types of pulses (soliton pulse, DSR, NLP, bunched soliton pulses, etc.) can exist in the harmonic mode-locking regime, in which the pulse reproduces itself with a multiplication of fundamental pulse repetition rate, further forming harmonic solitons [29], harmonic dissipative solitons [13], harmonic bunched solitons [27] and harmonic NLP [30]. Attractively, these pulses also can co-exist in a same fiber oscillator, which greatly enriches the ultrafast dynamics in a mode-locking laser. For example, near the zero-dispersion wavelength region of the glass fiber, two different SPs with non-equal pulse intensity are observed in an Er-doped fiber oscillator [31]. Additionally, in the Er-doped fiber oscillator, harmonic bunched-solitons and NLP are simultaneously achieved with a high nonlinear fiber [25]. On the other hand, fiber oscillators operating above the zero-dispersion wavelength region can provide a natural anomalous dispersion environment. These fiber oscillators including 2 μm thulium (Tm)-doped, holmium (Ho)-doped, or Tm–Ho-codoped fiber oscillators provide another platforms for the investigation of pulse evolution dynamics [32,33,34,35]. The large gain of the Tm-doped fiber mainly is located in the <2000 nm wavelength region, while with the assistance of Ho^3+^ ion, the large net-gain can be extended easily to the >2000 nm wavelength region in a Tm–Ho-codoped fiber, resulting in a broadband wavelength emission ranging from 1.7 μm to 2.1 μm. Besides that, the dual ions doped ultrafast fiber laser can provide more abundant pulse dynamics due to the interaction between co-doped ions. In this work, we fist report the coexistence phenomenon of NLP and SP in a nonlinear polarization rotation (NPR) mode-locking Tm–Ho-codoped fiber oscillator. The harmonic soliton pulse and NLP also can be obtained separately with proper design of the fiber cavity. In the co-existence regime, a maximum average output power of 295 mW is realized with a pulse repetition rate of 19.85 MHz, resulting in a pulse energy of 14.86 nJ. The dual-NLPs or SPs at two different wavelengths, or single NLP and SP at one wavelength, are also obtained respectively by adjusting the wave plates. Moreover, harmonic soliton mode-locking with 61-order pulse is also realized by increasing cavity length. The physical formation mechanism for the coexistence of different mode-locking pulses is analyzed.

## 2. Results and Discussion

**Figure 2:**
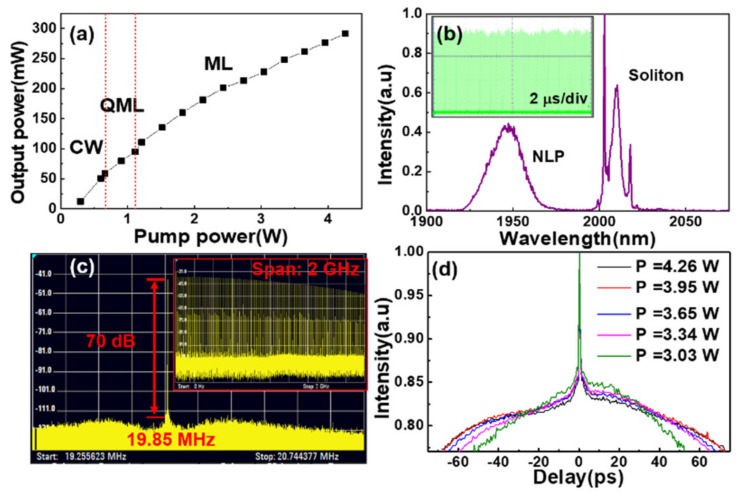
(**a**) Output power versus pump power. cw: continuous wave, QML: Q-switched mode-locking, ML: mode-locking. (**b**) Output laser spectrum in the NLP and SP coexisted mode-locking regime. Inset: typical mode-locking pulse train at the time scale of 20 μs. (**c**) The radio frequency spectral at the scanning ranges of 1.5 MHz. Inset: 2 GHz. (**d**) The pulse auto-correlation traces under different pump powers, P: pump power.

The NLP and SP coexisted mode-locking operation is realized by carefully adjusting wave plates at the pump power above 1.2 W. The power performance is recorded as Figure 2a. As the pump power scales up, the fiber laser gradually evolves from the continuous wave (cw) regime to the Q-switched mode-locking (QML) regime and finally to the dual-pulse coexisted mode-locking regime. The maximum average output power reaches to 292 mW at the pump power of 4.23 W. Figure 2b shows the spectrum of NLP locates at a short wavelength (~1947 nm) and possesses a smooth profile with a bandwidth of ~22 nm. The small spikes riding on the NLP spectrum are attributed to the absorption of water vapor in air. The long emission spectrum, which is the spectrum of SP verified by the symmetrically distributed Kelly sidebands, locates around ~2010 nm with a spectral bandwidth of about 5 nm. The typical mode-locking pulse train is shown as inset of Figure 2b, giving a pulse-to-pulse fluctuation of about 12%, which is deteriorated by the instable NLP mode-locking. For further estimating the stability of the mode-locking operation, the radio frequency (RF) spectrum is measured for different scanning ranges shown in Figure 2c. The RF spectrum of SP is overwhelmed by the RF spectrum of NLP, which possesses a wide width and two sidelobes at the bottom of the fundamental frequency. The fundamental frequency is 19.85 MHz with a signal-to-noise ratio (SNR) of 70 dB, matching well with the fiber cavity length. In a broad RF spectrum range up to 2 GHz, the RF spectrum (inset of Figure 2c) shows a broad comb of harmonics with a SNR higher than 40 dB. The pulse auto-correlation trace is also featured with the characteristic of the NLP, which consists of a narrow femtosecond spike and a hundred picoseconds pedestal (Figure 2d) [22,30]. The cross-section of the pedestal in the auto-correlation trace increases as the pump power scales up, implying the simultaneous increasing of the pulse energy of NLP.

**Figure 3:**
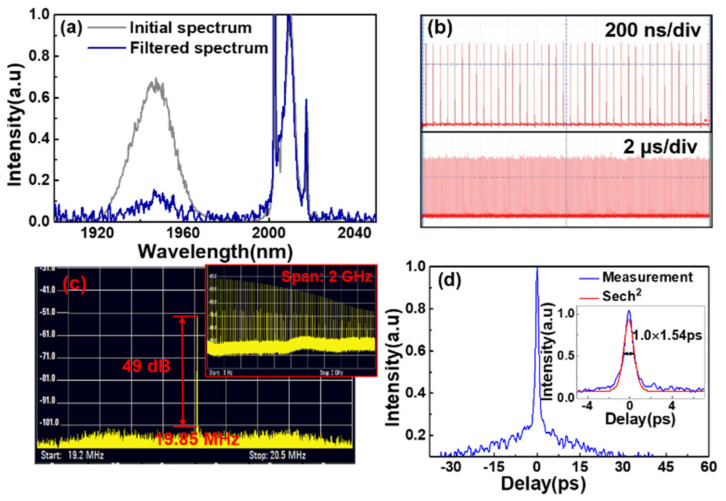
(**a**) The initial spectrum (gray line) and filtered spectrum (blue line). (**b**) The SP trains at the time scales of 2 and 20 μs. (**c**) The RF spectrum of SP at the scanning ranges of 1.3 MHz and 2 GHz (inset). (**d**) The SP auto-correlation trace. Inset: the SP auto-correlation trace fitted by the sech^2^ function.

**Figure 4:**
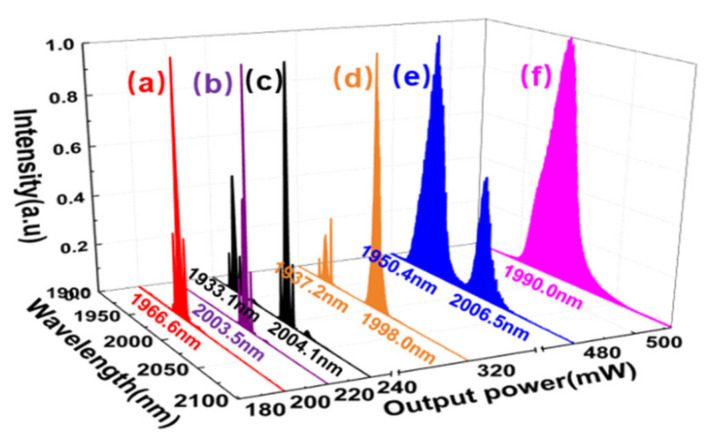
The output spectrum under different average output powers evolutions at the maximum pump power. The number marks the central wavelength of the emission spectrum. Single wavelength for single SP (**a**), (**b**) and single NLP (**f**), dual-wavelengths for dual-SPs (**c**) and dual-NLPs (**e**), and the coexisted SP and NLP (**d**).

In order to separately investigate the SP, a filter is utilized to move away the NLP in the short wavelength region (<2000 nm). The performances of the SP are shown in Figure 3. After the filter, the intensity of NLP is reduced remarkably, but the intensity of SP is almost unchanged relatively (see Figure 3a). Figure 3b shows the measured SP trains at the time scales of 2 μs and 20 μs. The pulse-to-pulse fluctuation is reduced from 12% (see Figure 2b) to 5%. The RF spectrum in Figure 3c shows the SNR of fundamental frequency is 49 dB and there is no obvious NLP induced sidelobes. The inset of Figure 3c indicates the SNR of the harmonic combs is still larger than 20 dB at the 2 GHz scanning range. The measured SP auto-correlation trace shown in Figure 3d has a pedestal with a ~20 ps duration, arising from the residual NLP (see Figure 3a). Assuming a sech^2^ pulse shape, the pulse duration is determined to be 1 ps (inset of Figure 3d). The time-bandwidth product is evaluated to be 0.353, approaching to the Fourier transformation limited value of 0.315. By carefully rotating wave plates at the maximum pump power, the SPs at one wavelength (1966.6 or 2003.5 nm) or dual-wavelengths (1933.1 and 2004.1 nm), NLPs at one wavelength (1990.0 nm) or dual-wavelengths (1950.4 and 2006.5 nm), and the coexisted SP and NLP at dual wavelengths (1937.2 and 1998.0 nm) are also observed as shown in Figure 4. The wavelength spacing of the dual center wavelengths is always around 60 nm in different mode-locking regimes. Among these regimes, the maximum average output power of 512 mW is realized for the single NLP mode-locking at 1990.0 nm, resulting in a pulse energy of 25.8 nJ.

**Figure 5:**
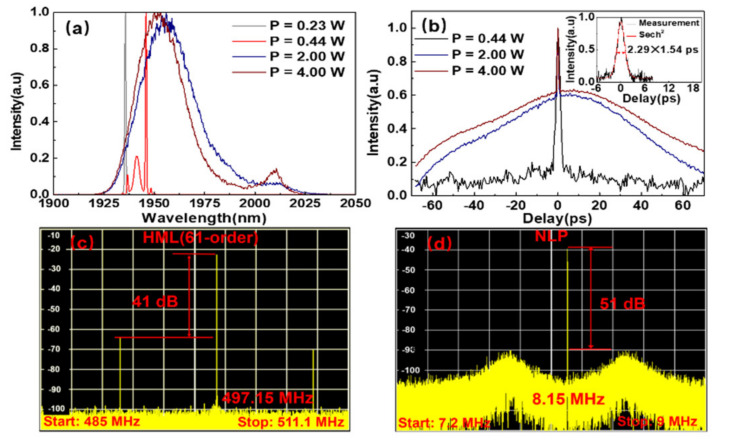
(**a**) The emission spectra under different pump powers. (**b**) The measured autocorrelation traces under different pump powers. Inset: the SP auto-correlation trace fitted by the sech^2^ function at 61-order HML regime. The RF spectra of the 61-order soliton (**c**) and the NLP (**d**) at the scanning range of 26.1 MHz and 1.8 MHz. HML: harmonic mode-locking.

By slightly changing the parameter of the laser cavity with increasing the cavity length to ~25.4 m, the soliton harmonic mode-locking and NLP mode-locking are also realized, respectively. As the pump power scales up from 0.23 W to 4 W, the fiber oscillator can operate in cw regime, soliton harmonic mode-locking (HML) regime and NLP mode-locking regime. These different regimes can be easily distinguished from the emission spectra, which are shown in Figure 5a. In the soliton HML regime, the pulse repetition rate can reach to 497.15 MHz, corresponding to the 61-order of the fundamental pulse repetition rate. This is the highest soliton order compared with the previously reported Tm–Ho-codoped HML fiber oscillators. The pulse duration is 2.29 ps by assuming a sech^2^ pulse shape (inset of Figure 5b) and RF spectrum shows a SNR of 41 dB (see Figure 5c). In NLP mode-locking regime, the dual-wavelengths with a spacing also around ~60 nm are observed, and the central wavelengths approach to that in Figure 2b. The broader pedestal of NLP in Figure 5b indicates a much higher pulse energy (33.4 nJ) than that in Figure 2d. The RF spectrum in Figure 5d shows more obvious sidelobes at the bottom of the fundamental frequency with a SNR of 51 dB. It should be noted that the limited resolution of the instruments for charactering ultrafast pulses, the output instability of the fiber oscillator itself, and the environmental fluctuations can result in some uncertainties for the measured ultrafast pulse performances. In addition, the phase noise uncertainty can be precisely measured as that investigated in Reference [36].

In the experiment, dual wavelength operations are always achieved in different mode-locking regimes. The dual wavelength operation could arise from the spectral filter effect in the birefringent fiber or the emissions of Tm^3+^ and Ho^3+^ ions in the Tm-Ho-codoped active fiber. The period of spectra filter Δ*λ* induced by birefringence can be expressed as [37]:

(1) $$\Delta \lambda =\frac{\lambda^{2}}{LB_{m}+n_{2}PLcos\left(2\theta \right)/A_{eff}}$$

where *λ* is the emission wavelength, *L* is the length of birefringent fiber, *B_m_* = *n_x_* − *n_y_* is the modal birefringence, *n_x_* and *n_y_* are the refractive index for different polarizations, *n_2_* is the nonlinear refractive index, *P* is the instantaneous power of the laser, *θ* is the angle depending on the rotation of wave-plates, and *A_eff_* is the effective mode area. In the experiment, the SMF can function as the birefringent fiber as Reference [37] so that the calculated modal birefringence *B_m_* is 4.9 × 10^−6^ and the nonlinear refractive index *n*_2_ is 2.7 × 10^−20^ m^2^/W [37]. According to the experimental results, we set *λ* = 1950 nm, *P* = 0.1 W, *A_eff_* = 254 µm^2^, and Δ*λ* ≈ 60 nm, while a proper *θ* is unable to be solved with the experimental cavity length *L* of 10.4 m or 25.4 m. Therefore, we believe that the dual-wavelength emission is independent of the spectral filter effect in the SMF-based birefringent fiber. Moreover, we find that the dual-wavelength emission only is observed when the pump power exceeds a certain value as shown in Figure 5. This is a main characteristic of Tm–Ho-co-doped laser which requires a high pump power for energy transfer between Tm^3+^ and Ho^3+^ ions to emit dual wavelengths. The formation mechanism for dual-wavelength emission is different from those reported methods [30,38,39,40,41,42,43,44].

**Figure 6:**
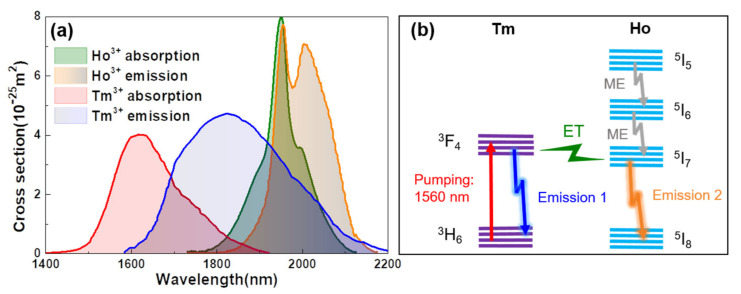
(**a**) The absorption and emission cross section of Tm^3+^ and Ho^3+^. (**b**) The simplified energy diagram of Tm–Ho-codoped gain fiber pumped at 1560 nm. ET: energy transition, ME: multi-phonon emission.

The emission and absorption spectra of Tm^3+^ and Ho^3+^ ions are shown in Figure 6a. Although the gain wavelength region of the Tm^3+^ ion is partly overlapped with the absorption of Ho^3+^ ion, there still exists net gain in the wavelength region below 2000 nm. With the assistance of Ho^3+^ ion, the large net-gain can be extended to above 2000-nm wavelength region in a Tm–Ho-codoped system. The ion transition processes in the Tm–Ho-codoped active fiber are simplified as shown in Figure 6b. The Tm^3+^ ions at the ground state of ^3^H_6_ are excited to the upper energy level ^3^F_4_ by the 1560-nm pumping laser. When the pump power is low, most of the Tm^3+^ ions at the energy level of ^3^F_4_ will return to the ^3^H_6_ accompanied by the laser emission in the short wavelength region below 2000 nm (laser emission 1 in Figure 6b). The energy transition between ^3^F_4_ in Tm^3+^ ion and ^5^I_7_ in Ho^3+^ ion can be ignored so that only one wavelength emission can be observed under weak pump power. As the pump power scales up, the energy level of ^3^F_4_ in the Tm^3+^ ion is strongly occupied, which results in a large energy transition between Tm^3+^ ion and Ho^3+^ ion. So other than the laser emission in short wavelength region, the transition from the energy level ^5^I_7_ to the energy level ^5^I_8_ of Ho^3+^ ions generates another laser emission above 2000 nm (laser emission 2 in Figure 6b). It should be noted that for the coexisted pulses at dual wavelengths, the laser emission in short wavelength region always possesses a large gain compared with that in the long wavelength region due to the low concentration of Ho^3+^ ions. For example, as shown in Figure 2b, Figure 4e and Figure 5a, under strong pump power, the SP or NLP with a low pulse energy is formed at a long wavelength via the emission transition in Ho^3+^ ion, while the formed pulse at short wavelength accumulates a large energy due to the large gain, which facilities the formation of large energy NLP. However, in Figure 4b–d, we find that the SP and NLP with large energies are also formed around 2000 nm. We believe this is attributed to the co-interaction of Tm^3+^ and Ho^3+^ ions in this wavelength region (see Figure 6a). 

## 3. Materials and Methods 

**Figure 7:**
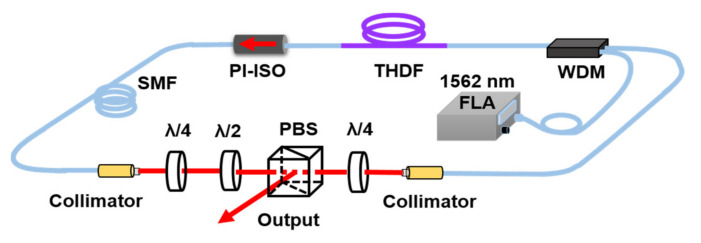
Schematic diagram of the Tm–Ho-codoped fiber oscillator. FLA: fiber laser amplifier; PI-ISO: polarization-independent isolator; WDM: wavelength division multiplex; THDF: Tm–Ho-codoped fiber; SMF: single-mode-fiber; PBS: polarization beam splitter; λ/2: half-wave plate; λ/4: quarter-wave plate.

The schematic diagram of the Tm–Ho-codoped mode-locking fiber oscillator is shown as Figure 7. The pump source is a continuous wave 1562-nm Er-doped fiber laser amplifier (FLA), which delivers a maximum output power of 4.23 W with a power instability of 0.3% measured within 60 min. The pumping laser is guided into the Tm–Ho-codoped fiber ring cavity by a wavelength division multiplex (WDM). The fiber ring cavity consists of a 4.3 m long Tm–Ho-codoped single mode active fiber (Coractive, SM-TH512, 23 dB/m at 1570 nm, −56 ps^2^/km at 1900 nm, CAN), a polarization independent isolator (PI-ISO), a group of NPR free-space optical components, and a 5.4 m long passive single-mode fiber (Nufern, SMF-28e, −67 ps^2^/km at 1900 nm, USA). The NPR optical component includes two quarter-wave plates, a half-wave plate, and a polarization beam splitter (PBS). The PBS simultaneously functions as both polarizer and output coupler. Considering the pigtail fiber of all optical components inside the fiber cavity, the total length of the fiber ring cavity is close to 10.4 m. The mode-locking operation can be realized by carefully adjusting the wave plates. The output pulse train is detected by an InGaAs PIN detector (EOT, ET-5000, USA) and observed with an oscilloscope (Tektronix, DPO 4102B-L, USA).

## 4. Conclusions

In this work, first we observe the coexisted noise-like pulse and soliton pulse in the thulium–holmium-codoped ultrafast fiber oscillator. By carefully adjusting the wave plates, the coexisted noise-like pulse and soliton pulse can involve into the formation of dual-noise-like pulses or dual-soliton pulses at dual-wavelengths and single noise-like pulse or single soliton pulse at one wavelength. A 61-order harmonic soliton pulse and the 33.4-nJ-noise-like pulse are also realized respectively by prolonging the fiber oscillator length. We believe the dual-wavelength emissions are attributed to the transitions of Tm^3+^ and Ho^3+^ ions respectively of the gain fiber. The coexisted NLP and SP at different wavelengths depend on the different gain under a strong pumping power. 

## Data Availability

Data are contained within this article.

## Sample Availability

Samples of the patches are available from the authors.
